# Beyond the Bones: Primary Prostatic Synovial Sarcoma in a Young Adult

**DOI:** 10.7759/cureus.116392

**Published:** 2026-09-16

**Authors:** Thalapathi Raja, Rubina Singh, Venkateshen Palanisamy, Natarajan Kumaresan, Chandru T

**Affiliations:** 1 Urology, Sri Ramachandra Institute of Higher Education and Research, Chennai, IND; 2 Urology and Renal Transplantation, Sri Ramachandra Institute of Higher Education and Research, Chennai, IND

**Keywords:** case report, chemotherapy, pelvic mass, prostate, soft tissue sarcoma, synovial sarcoma

## Abstract

Synovial sarcoma is a very rare, highly malignant mesenchymal tumor and predominantly affects adolescents and young adults. This neoplasm tends to develop near the large joints in the extremities. The incidence of this type of neoplasm involving the prostate gland is extremely rare. Prostate sarcoma can often be misdiagnosed as a consequence of its infrequent occurrence. This is a case presentation of a 37-year-old man who had complained of a weak urinary stream for three weeks. Digital rectal examination showed an enlarged, hard, and nodular prostate. On imaging, there was a huge heterogeneous pelvic mass that was located behind the urinary bladder and was suspected to be originating from the prostate gland with cystic and necrotic components. A transrectal ultrasound-guided biopsy showed a diagnosis of primary synovial sarcoma of the prostate. Chemotherapy using doxorubicin (Adriamycin) and cyclophosphamide was administered. Primary prostatic synovial sarcoma represents an exceptionally rare entity with diagnostic and therapeutic challenges. Early recognition, histopathological confirmation, and multidisciplinary treatment may improve outcomes in this aggressive malignancy.

## Introduction

Synovial sarcoma is an uncommon, highly aggressive malignant mesenchymal tumor that accounts for approximately 5-10% of soft tissue sarcomas and predominantly affects adolescents and young adults [1]. It most commonly arises in para-articular regions of the extremities, although, despite its name, it does not originate from the synovium. Extra-skeletal synovial sarcoma can occur in unusual anatomical sites, including the heart, lungs, kidneys, retroperitoneum, alimentary tract, and genitourinary system [2]. Primary involvement of the prostate is exceptionally rare, with only a limited number of cases documented in the literature [3].

Primary prostatic synovial sarcoma may present with nonspecific lower urinary tract symptoms, including weak urinary stream, dysuria, urinary retention, hematuria, or pelvic pain resulting from a bulky pelvic mass [4,5]. These manifestations may resemble more common prostatic conditions such as benign prostatic hyperplasia, prostatitis, or prostatic adenocarcinoma, potentially delaying diagnosis. Imaging typically demonstrates a large heterogeneous mass with cystic degeneration, necrosis, or hemorrhage, but these findings are not specific. Histopathological assessment supported by immunohistochemistry is therefore essential, while molecular evaluation for the characteristic t(X;18)(p11.2;q11.2) translocation resulting in SS18-SSX fusion can provide additional diagnostic confirmation when available [2,6].

Because primary prostatic synovial sarcoma is extremely uncommon, evidence regarding its optimal diagnostic and therapeutic management remains limited, and standardized treatment guidelines have not been established [3,7,8]. Management is generally extrapolated from the treatment of soft tissue synovial sarcoma and may involve surgery, chemotherapy, and radiotherapy, depending on tumor extent and clinical circumstances. The present case highlights the diagnostic challenge posed by a large prostatic/pelvic mass in a young adult presenting primarily with obstructive urinary symptoms and emphasizes the importance of considering rare mesenchymal malignancies in the differential diagnosis of atypical prostatic masses.

## Case presentation

A 37-year-old gentleman with complaints of weak urine flow for three weeks was seen. His past history included a lateral internal sphincterotomy done two months ago. There was no history of hematuria, fever, or weight loss. Physical examination was essentially normal. On systemic examination, the abdomen was soft and non-tender without palpable organomegaly. External genital examination showed no phimosis, and bilateral groins were normal. Digital rectal examination revealed an enlarged, hard, and nodular prostate. Routine laboratory investigations demonstrated a hemoglobin of 14.7 g/dL and a total leukocyte count of 8490 cells/mm³. Renal function parameters were within normal limits. Scrotal ultrasonography demonstrated increased vascularity in the right testis suggestive of orchitis with a right epididymal cyst measuring approximately 7×6 mm. The left testis was noted to be small in size, positioned relatively higher within the scrotum, with diffuse heteroechoic echotexture, tiny calcifications, and reduced internal vascularity, suggestive of chronic left testicular torsion with testicular atrophy.

Figure 1 represents the ultrasonography of the kidney-ureter-bladder region revealing a well-defined heteroechoic pelvic mass located posterior to the urinary bladder measuring approximately 7.8×9.8×10.8 cm with an estimated volume of 412 cc. The lesion appeared predominantly solid with few cystic areas, thick internal septations, and minimal internal vascularity. The prostate and seminal vesicles could not be visualized separately. 

**Figure 1:**
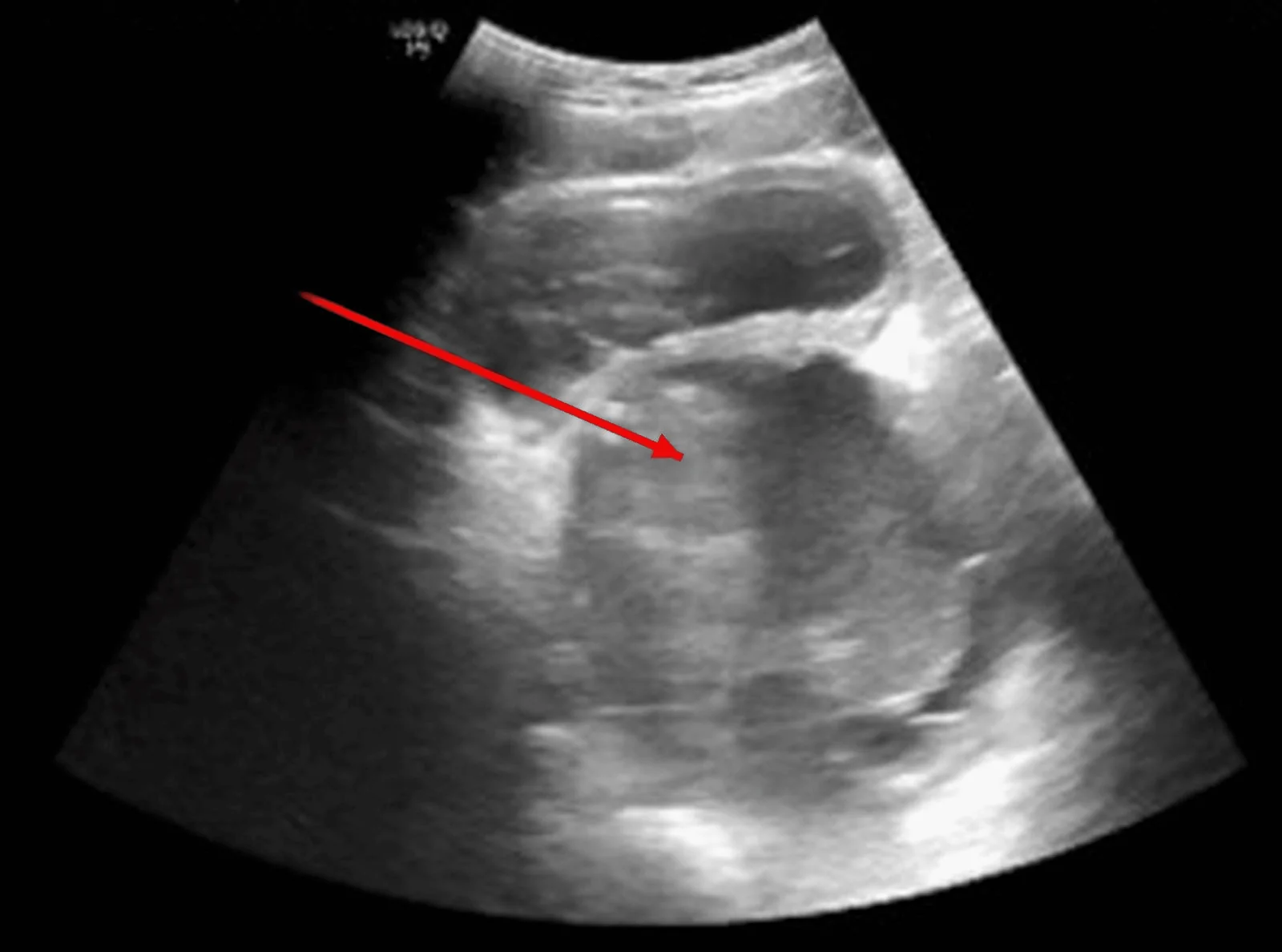
Ultrasonography of the kidney-ureter-bladder region showing a heteroechoic pelvic mass posterior to the urinary bladder → denotes the pelvic mass.

Further contrast-enhanced imaging demonstrated a well-defined heterogeneously enhancing pelvic mass lesion with non-enhancing cystic and necrotic areas arising posterior to the urinary bladder, with probable epicenter involving the posterior transition and peripheral zones of both prostatic lobes. The lesion measured approximately 8×8.9×7.4 cm and exerted mass effect on adjacent structures. Contrast-enhanced computed tomography (CECT) of the abdomen and pelvis demonstrated a large, bulky, heterogeneous pelvic mass arising from the prostate (Figure 2). The coronal image (Figure 2A) demonstrated a bulky pelvic lesion extending superiorly into the lower abdomen. The sagittal image (Figure 2B) showed the mass located posterior to the urinary bladder, with significant compression and displacement of adjacent pelvic structures. The axial image (Figure 2C) demonstrated marked heterogeneous attenuation with areas of low attenuation suggestive of cystic/necrotic changes. Overall, the imaging features were concerning for an aggressive soft tissue neoplasm, with prostatic sarcoma considered in the differential diagnosis. 

**Figure 2:**
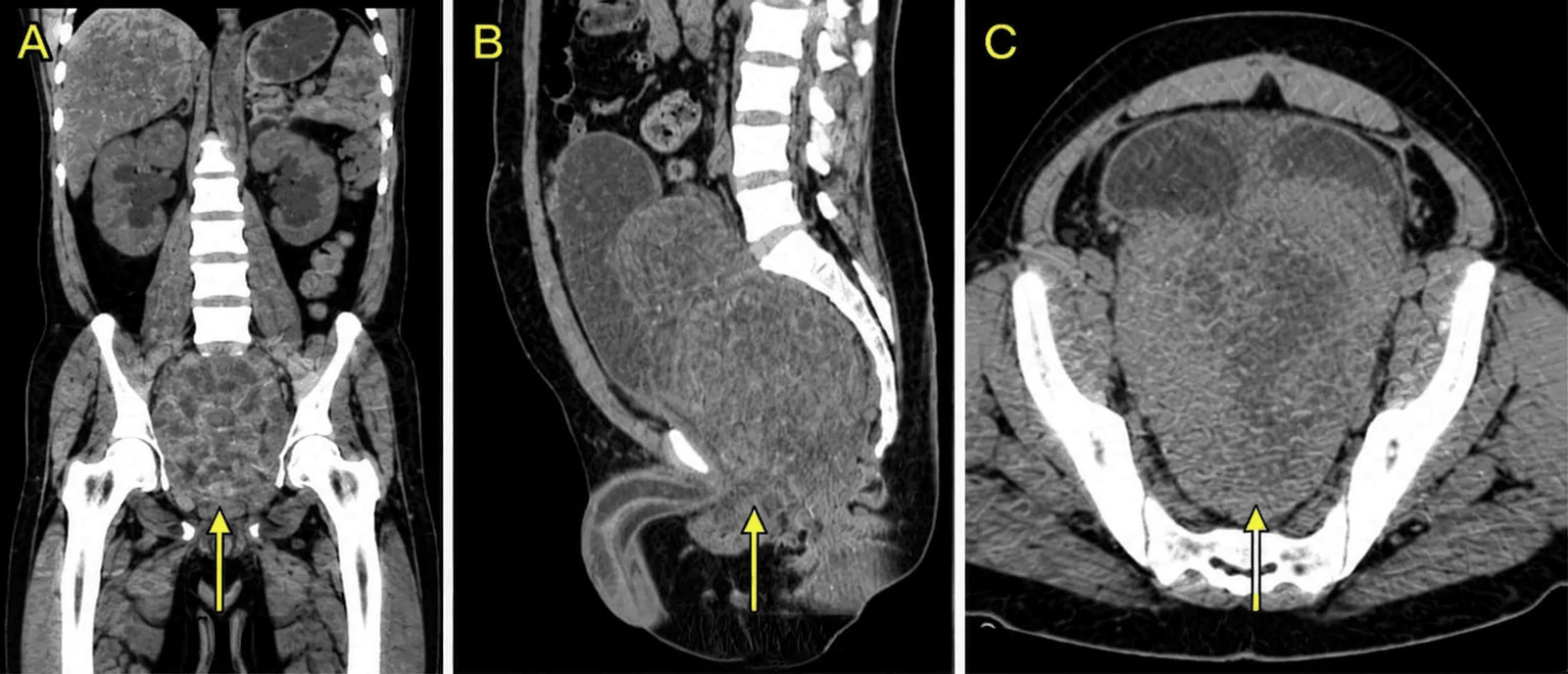
Contrast-enhanced computed tomography of the abdomen and pelvis showing a large heterogeneous prostatic mass (A) Coronal image demonstrating a bulky pelvic lesion. (B) Sagittal image showing the mass located posterior to the urinary bladder with compression of adjacent structures. (C) Axial image demonstrating heterogeneous attenuation with cystic/necrotic areas suggestive of a soft tissue sarcoma. → denotes the pelvic mass.

Transrectal ultrasound-guided sextant biopsy was performed. Histopathological examination confirmed the diagnosis of primary synovial sarcoma of the prostate in Figure 3. Histopathological examination of the prostatic mass revealed a spindle cell neoplasm arranged predominantly in fascicles, with moderate nuclear pleomorphism and scant cytoplasm on hematoxylin and eosin (H&E) staining (Figure 3A). Immunohistochemical evaluation demonstrated strong diffuse nuclear positivity for TLE1 in the tumor cells, whereas the adjacent benign prostatic glands were negative (Figure 3B). Further immunohistochemical staining for SS18-SSX showed diffuse and strong nuclear positivity in the tumor cells (Figure 3C). These histopathological and immunohistochemical findings supported the diagnosis of primary prostatic synovial sarcoma.

**Figure 3:**
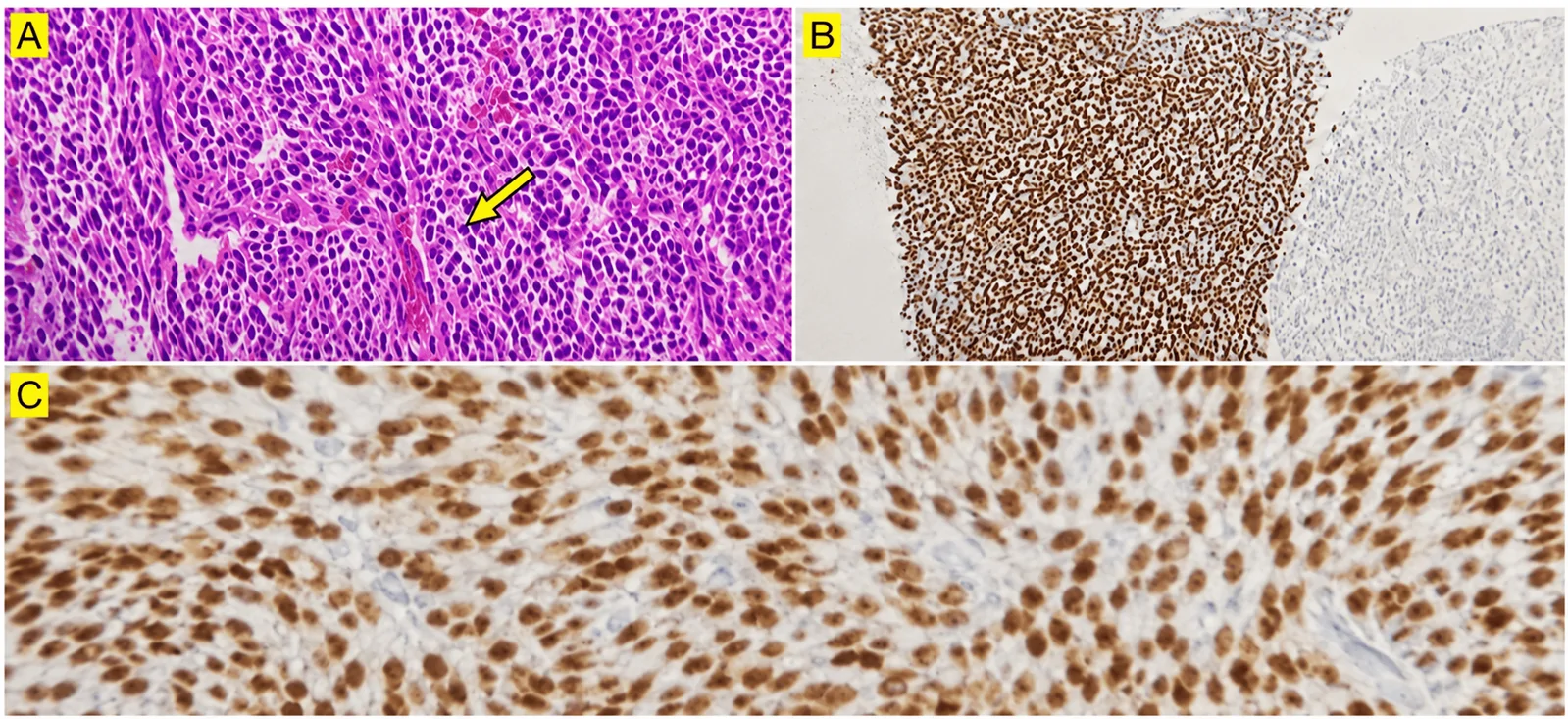
Histopathological and immunohistochemical findings in primary prostatic synovial sarcoma (A) Hematoxylin and eosin-stained section showing a densely cellular malignant spindle cell neoplasm composed of spindle-to-ovoid cells with scant cytoplasm and hyperchromatic nuclei; the arrow denotes the spindle cell proliferation. Areas of hemorrhage and atypical mitotic activity are present. (B) Low-power immunohistochemistry demonstrating diffuse strong nuclear positivity for TLE1. (C) High-power view confirming diffuse nuclear TLE1 expression in tumor cells. The immunoprofile showed negativity for CD117, desmin, NKX2.2, and cytokeratin, with a Ki-67 labeling index of approximately 20%. SS18-SSX immunohistochemical staining demonstrated strong diffuse nuclear positivity. Molecular confirmation of an SS18-SSX rearrangement by fluorescence in situ hybridization was advised but was not performed because of financial constraints and limited availability.

Figure 4 presents the fluorine-18 fluorodeoxyglucose (18F-FDG) positron emission tomography (PET)/CT findings in primary prostatic synovial sarcoma. Anterior coronal PET images (Figure 4A-4C) demonstrate intense and focal 18F-FDG uptake corresponding to the prostatic mass. The uptake remains prominent on the delayed image, indicating high metabolic activity of the lesion. No definite abnormal FDG-avid regional lymph nodes or distant metastatic lesions are identified, suggesting a localized primary prostatic malignancy. In the context of the patient's young age and subsequent histopathological and immunohistochemical findings, the imaging features were consistent with primary prostatic synovial sarcoma.

**Figure 4:**
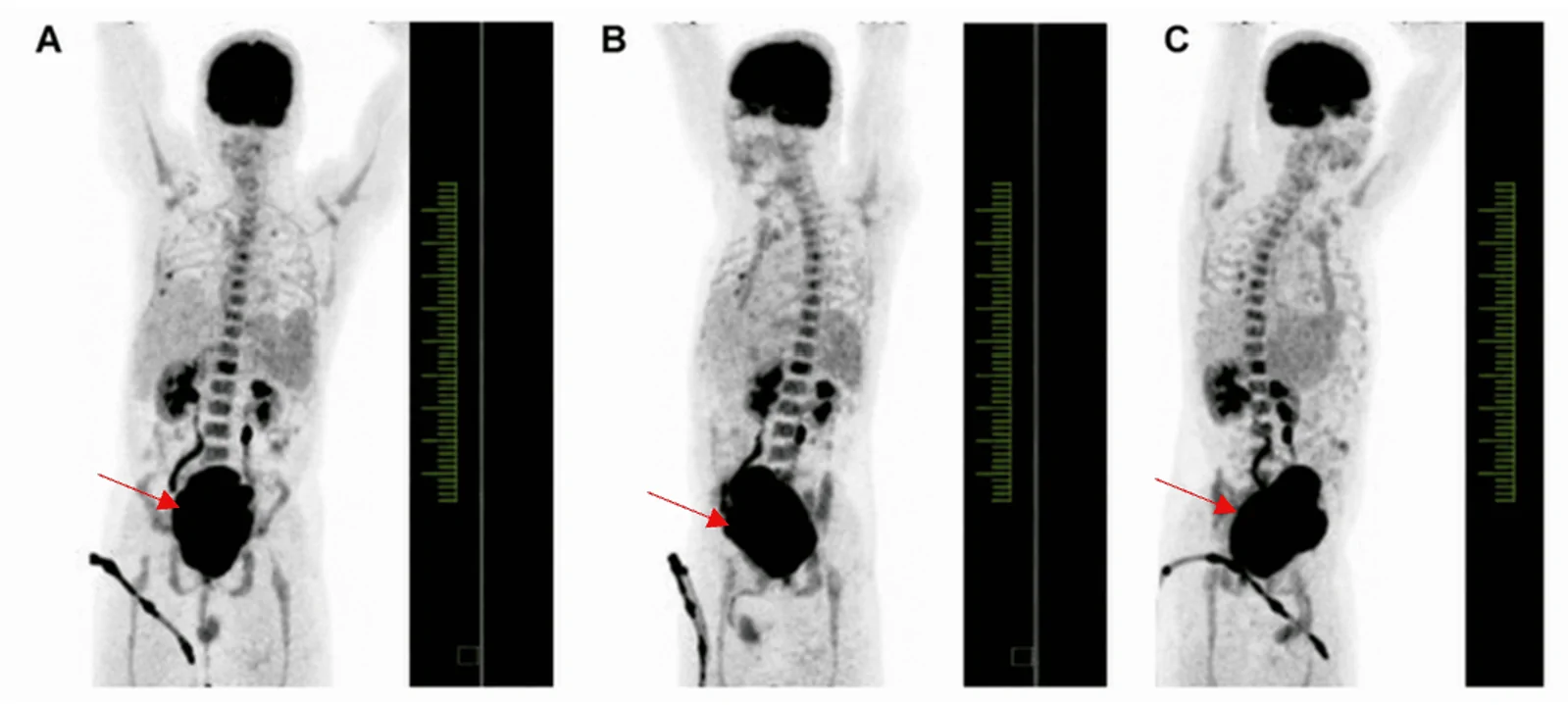
18F-FDG PET imaging of primary prostatic synovial sarcoma (A) Anterior coronal 18F-FDG PET image demonstrating focally increased FDG uptake in the prostate region, raising suspicion for a primary prostatic neoplasm. (B) PET image showing intense FDG uptake localized to the prostate, without evidence of significant regional lymph node involvement or distant metastatic disease. (C) Delayed anterior coronal PET image demonstrating persistent intense FDG uptake within the prostatic lesion, supporting the presence of a metabolically active primary prostatic tumor. In the clinical context, these findings were consistent with primary prostatic synovial sarcoma in a young adult. → denotes the primary FDG-avid prostatic lesion. 18F-FDG: fluorine-18 fluorodeoxyglucose; PET: positron emission tomography

Contrast-enhanced CT of the pelvis revealed a large, well-defined soft tissue mass arising from the prostate, resulting in marked enlargement of the gland and demonstrating heterogeneous enhancement. 18F-FDG PET showed intense focal FDG uptake corresponding to the prostatic mass, indicating increased metabolic activity. Fused PET/CT images demonstrated clear anatomical correlation between the enhancing prostatic lesion and the area of intense FDG uptake. No significant FDG-avid regional lymphadenopathy or distant metastatic lesions were identified, suggesting a localized primary prostatic malignancy. In view of the patient's young age, radiological characteristics, and subsequent histopathological and immunohistochemical findings, the lesion was diagnosed as primary prostatic synovial sarcoma (Figures 5-6). The patient subsequently received doxorubicin (Adriamycin) and cyclophosphamide-based chemotherapy. 

**Figure 5:**
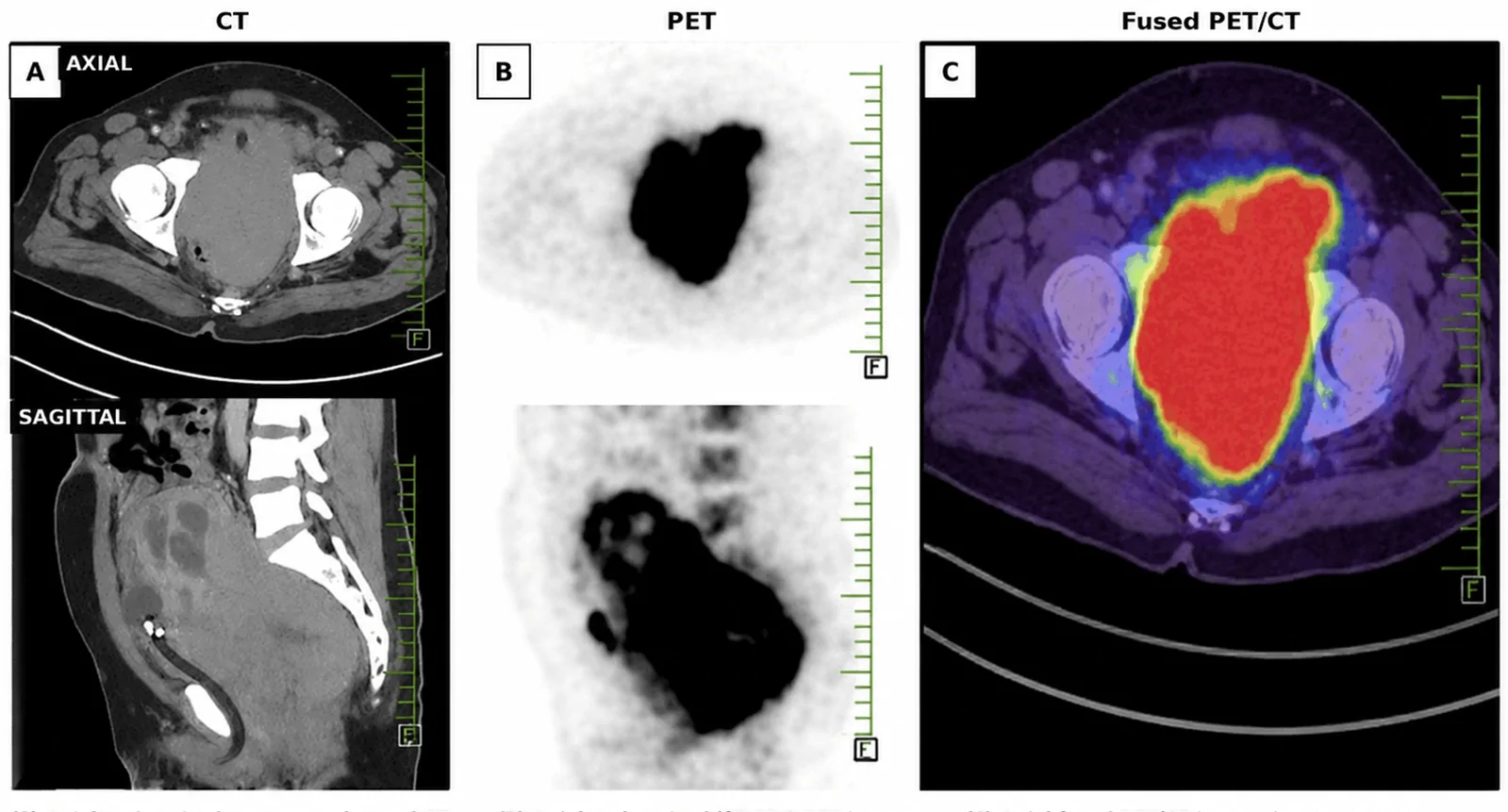
Axial and sagittal PET views of the primary pelvic mass (A) Axial and sagittal contrast-enhanced CT images demonstrating a well-defined soft tissue mass arising from the prostate, causing marked enlargement of the gland with heterogeneous enhancement. (B) Axial and sagittal 18F-FDG PET images demonstrating intense focal FDG uptake corresponding to the prostatic mass, indicating high metabolic activity. (C) Fused PET/CT images demonstrating intense FDG uptake localized to the prostatic lesion, confirming its metabolic activity and anatomical correlation with the mass. No significant FDG-avid regional lymph node involvement or distant metastatic lesions are identified, suggesting a localized primary prostatic malignancy. In the clinical and histopathological context, these imaging findings are consistent with primary prostatic synovial sarcoma. 18F-FDG: fluorine-18 fluorodeoxyglucose; PET: positron emission tomography; CT: computed tomography

**Figure 6:**
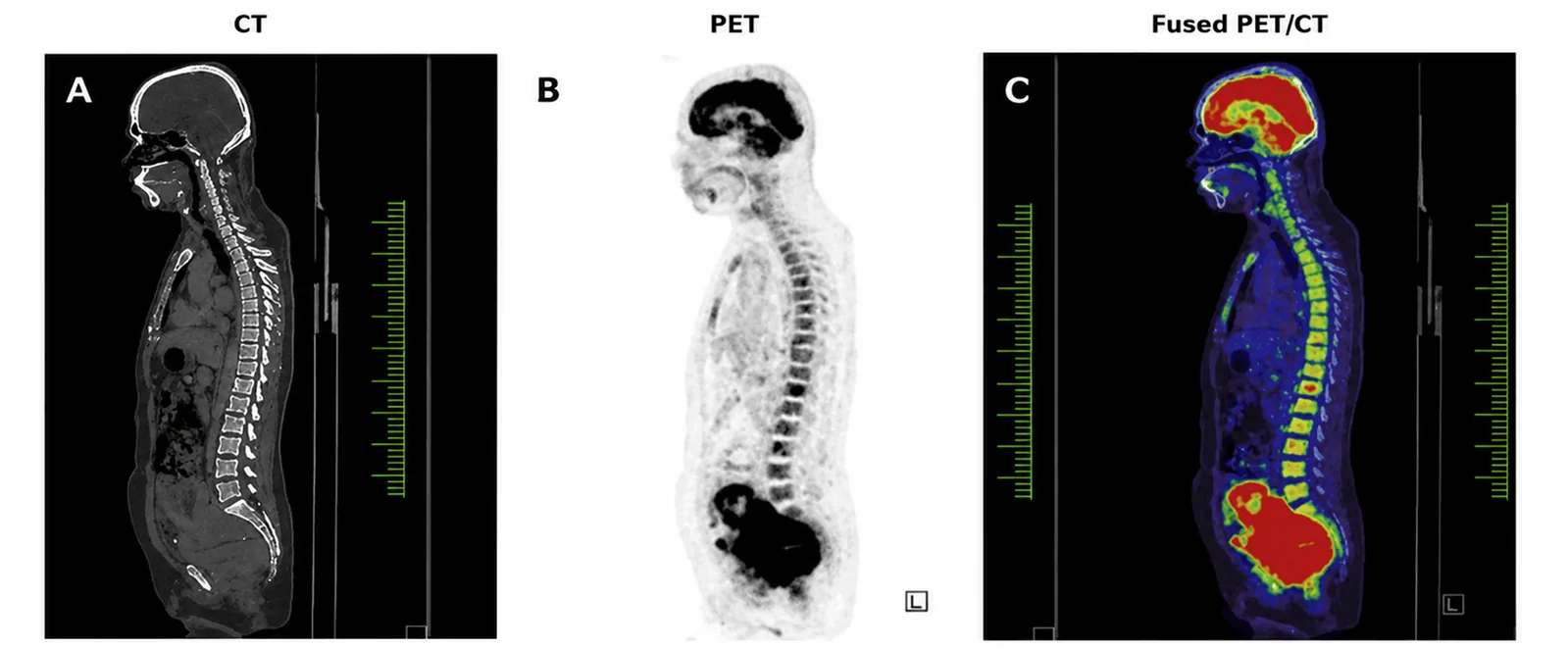
Sagittal PET profiling spinal metastasis and urinary tract obstruction (A) Sagittal contrast-enhanced CT image demonstrating a well-defined soft tissue mass arising from the prostate, causing marked enlargement of the gland with heterogeneous enhancement and no obvious extracapsular extension. (B) Sagittal 18F-FDG PET image demonstrating intense focal FDG uptake corresponding to the prostatic mass, indicating increased metabolic activity. (C) Sagittal fused PET/CT image demonstrating intense FDG uptake localized to the prostatic lesion, with good anatomical correlation with the mass identified on CT. No significant FDG-avid regional lymph node involvement or distant metastatic disease is identified. These findings are consistent with a metabolically active localized primary prostatic malignancy and, in the appropriate clinical and histopathological context, support the diagnosis of primary prostatic synovial sarcoma. 18F-FDG: fluorine-18 fluorodeoxyglucose; PET: positron emission tomography; CT: computed tomography

At the time of evaluation, the patient's serum prostate-specific antigen (PSA) level was within the normal range. Other tumor markers, including beta-human chorionic gonadotropin (beta-hCG), alpha-fetoprotein (AFP), and lactate dehydrogenase (LDH), were not performed and therefore were not available for the evaluation of the prostatic/pelvic mass or exclusion of other potential malignant diagnoses. Histopathological examination demonstrated a malignant spindle cell neoplasm, with strong diffuse nuclear positivity for TLE1 and SS18-SSX immunohistochemical staining. The immunohistochemical panel was negative for CD117, desmin, NKX2.2, and cytokeratin. Molecular confirmation of an SS18-SSX rearrangement by fluorescence in situ hybridization (FISH) was advised but was not performed because of financial constraints and limited availability of the test at the treating institution. Thus, the diagnosis was based on the characteristic morphological and immunohistochemical findings, with the absence of molecular confirmation acknowledged as a limitation. Baseline 18F-FDG PET/CT demonstrated a large prostatic/pelvic mass without significant FDG-avid regional lymphadenopathy or distant metastatic disease. Given the rarity of primary prostatic synovial sarcoma and the absence of a validated site-specific staging system for this tumor, a formal disease-specific TNM stage was not assigned. The patient's Eastern Cooperative Oncology Group (ECOG) performance status at treatment initiation was 1. These baseline clinical findings were considered in determining the subsequent treatment strategy.

Surgical treatment was considered; however, the patient was not willing to undergo surgery. In addition, the large size and locally extensive nature of the prostatic/pelvic tumor made definitive surgical resection technically challenging and potentially associated with substantial morbidity and functional consequences. These considerations were particularly relevant given the patient's young age. Radiotherapy was considered during multidisciplinary evaluation but was not pursued as the initial definitive local treatment because of the tumor's size, location, and locally extensive nature, with systemic chemotherapy selected as the initial treatment strategy. This approach was undertaken in the context of the extreme rarity of primary prostatic synovial sarcoma and the absence of standardized site-specific treatment guidelines. Therefore, systemic chemotherapy with doxorubicin (Adriamycin) and cyclophosphamide was selected as the initial treatment approach, with subsequent imaging demonstrating an interval reduction in tumor burden. An anthracycline-based regimen consisting of doxorubicin (Adriamycin) and cyclophosphamide was selected because anthracycline-based chemotherapy is an established systemic treatment approach for aggressive soft tissue sarcomas, including synovial sarcoma. Given the extreme rarity of primary prostatic synovial sarcoma and the absence of site-specific treatment guidelines, the therapeutic approach was extrapolated from established principles for soft tissue synovial sarcoma. The patient was discussed at a multidisciplinary tumor board. The participating specialties included urology, medical oncology, radiation oncology, radiology, and pathology. The patient received doxorubicin (Adriamycin) at 60 mg/m² intravenously plus cyclophosphamide at 600 mg/m² intravenously every 21 days, with six cycles planned. After three cycles, an interim 18F-FDG PET/CT was performed and demonstrated a marked interval reduction in the size and overall tumor burden of the primary prostatic/pelvic mass, indicating a favorable radiological response. The patient subsequently completed all six planned cycles of chemotherapy and remained under clinical and radiological surveillance. He was evaluated at a specialized sarcoma referral center. The case was reviewed by a multidisciplinary sarcoma team, and management was planned accordingly. A formal Response Evaluation Criteria in Solid Tumors (RECIST) 1.1 response assessment could not be performed because complete post-treatment target-lesion measurements were unavailable. At the latest available follow-up, no newly developed regional lymphadenopathy or distant metastatic disease was documented on the available imaging, indicating a favorable treatment response. The patient remains under close surveillance and follow-up.

## Discussion

Primary prostatic synovial sarcoma is an exceptionally rare and aggressive mesenchymal neoplasm occurring predominantly in young adults. Previously reported cases commonly describe nonspecific lower urinary tract symptoms, including dysuria, urinary obstruction, weak urinary stream, hematuria, or pelvic discomfort, often associated with a large pelvic or prostatic mass [2-5]. In the present case, the 37-year-old patient presented with poor urinary stream and was found to have a large heterogeneous prostatic/pelvic mass measuring approximately 8×8.9×7.4 cm, with cystic and necrotic components. Similar large heterogeneous masses and obstructive urinary symptoms have been described by Williams et al., Jun et al., and Hou et al. [3-5]. Thus, the clinical and radiological presentation was broadly consistent with the previously reported spectrum of primary prostatic synovial sarcoma (Table 1).

**Table 1:** Previously reported cases of primary prostatic synovial sarcoma and comparison with the present case

Study	Age (years)	Clinical presentation	Tumor characteristics	Treatment	Outcome
Williams et al. (2004) [4]	37	Obstructive urinary symptoms	Large prostatic mass	Multimodal treatment including surgery	Follow-up reported
Jun et al. (2008) [5]: Case 1	22	Dysuria and urinary obstruction	Large pelvic/prostatic mass	Surgery + adjuvant therapy	Recurrence reported
Jun et al. (2008) [5]: Case 2	46	Lower urinary tract symptoms	Large prostatic mass	Multimodal treatment	Follow-up reported
Hou et al. (2022) [3]	29	Dysuria and urinary frequency	Large heterogeneous prostatic lesion	Surgery + chemotherapy	Disease control during follow-up
Present case	37	Poor urinary stream	8×8.9×7.4 cm heterogeneous pelvic mass with cystic and necrotic components	Adriamycin + cyclophosphamide chemotherapy	Interval tumor reduction

Diagnostic challenges

The diagnosis of primary prostatic synovial sarcoma remains challenging because its clinical and radiological findings are nonspecific and may overlap with more common prostatic or pelvic neoplasms. Imaging may demonstrate a large heterogeneous mass with cystic degeneration, necrosis, hemorrhage, or local mass effect but cannot establish the histological diagnosis [2,3]. In this case, the lesion was initially identified as a bulky pelvic mass, and subsequent contrast-enhanced imaging suggested a prostatic epicenter. The normal serum PSA level in a young patient with a disproportionately large prostatic mass further supported the consideration of a non-epithelial malignancy rather than conventional prostatic adenocarcinoma.

Histopathological examination was therefore essential. The tumor demonstrated malignant spindle cell morphology with strong diffuse nuclear TLE1 expression, while the immunohistochemical panel was negative for CD117, desmin, NKX2.2, and cytokeratin. SS18-SSX immunohistochemical staining also demonstrated strong diffuse nuclear positivity. TLE1 and SS18-SSX immunohistochemical expression provided supportive diagnostic evidence; however, molecular confirmation of an SS18-SSX rearrangement by FISH was advised but could not be performed because of financial constraints and limited availability at the treating institution [9]. Therefore, the diagnosis was based on the characteristic morphological and immunohistochemical findings, while the absence of molecular confirmation is acknowledged as a limitation.

Treatment considerations

Complete surgical resection with acceptable functional morbidity is an important consideration when technically feasible. In the present case, surgery was considered but was not performed because the patient was unwilling to undergo surgery. In addition, the large size and locally extensive nature of the tumor made definitive resection technically challenging and potentially associated with substantial morbidity and functional consequences, particularly in this young patient.

Radiotherapy was considered during multidisciplinary evaluation but was not pursued as the initial definitive local treatment because of the tumor's large size, pelvic location, and locally extensive nature, together with the decision to proceed initially with systemic chemotherapy. Consequently, treatment was directed toward systemic chemotherapy with doxorubicin and cyclophosphamide. The patient received doxorubicin (Adriamycin) 60 mg/m² intravenously and cyclophosphamide 600 mg/m² intravenously every 21 days, with six cycles planned. This regimen was selected because anthracycline-based chemotherapy is an established systemic treatment option for aggressive soft tissue sarcomas, including synovial sarcoma, although evidence specific to the prostatic site remains limited [7,10-12].

An interim 18F-FDG PET/CT after three cycles demonstrated a marked reduction in tumor burden, suggesting a favorable radiological response. The patient subsequently completed six cycles and remained clinically stable on surveillance. However, because complete post-treatment tumor dimensions and quantitative SUVmax values were unavailable, a formal RECIST 1.1 response assessment could not be performed. The observed response should therefore be described as an interval radiological response rather than a RECIST-defined partial response.

Prognosis and survival

The prognosis of primary prostatic synovial sarcoma remains difficult to define because of its extreme rarity and the predominance of case reports and small series. Prognostic information derived from synovial sarcoma at other anatomical sites suggests that tumor size, local extent, histological characteristics, metastatic disease, and treatment response may influence outcome [7,9,11-13]. Larger tumors, extensive local invasion, and metastatic disease are generally associated with poorer outcomes, whereas localized disease and favorable treatment response may be associated with improved disease control [7,12].

The present patient had a large primary tumor but no significant FDG-avid regional lymphadenopathy or distant metastatic disease at baseline and subsequently demonstrated an interval reduction in tumor burden following chemotherapy. These findings are favorable in terms of disease control but do not permit the prediction of long-term survival. Published reports of primary prostatic synovial sarcoma have described local recurrence and metastatic progression, emphasizing the aggressive biological behavior of this tumor [2,3,5]. Therefore, the absence of metastatic disease at presentation should not be interpreted as evidence of a low-risk tumor.

Long-term clinical and radiological surveillance remains important because of the potential for local recurrence and distant metastasis. Reliable disease-specific survival estimates for primary prostatic synovial sarcoma cannot currently be established from the available literature.

Learning points

This case highlights several important clinical considerations. First, a large prostatic or pelvic mass in a young adult presenting with obstructive urinary symptoms should raise suspicion for uncommon mesenchymal malignancies, including synovial sarcoma. Second, imaging findings alone are insufficient for diagnosis, and histopathological evaluation supported by an appropriate immunohistochemical panel is essential. TLE1 and SS18-SSX immunohistochemistry may provide important diagnostic support, while molecular confirmation of the SS18-SSX fusion is desirable when available [6,9,11]. Third, treatment should be individualized through multidisciplinary sarcoma team evaluation because standardized management guidelines for primary prostatic synovial sarcoma are lacking. Surgery remains an important consideration when complete resection is feasible, whereas systemic chemotherapy may be considered when definitive local treatment is not possible or is declined [7,10,12]. Finally, the interval radiological response observed in this patient illustrates that tumor reduction may occur with anthracycline-based chemotherapy; however, longer follow-up is required before any conclusion regarding durable disease control or survival can be made. 

## Conclusions

Prostate primary synovial sarcoma is an extremely uncommon disease with a high degree of aggressiveness, usually manifesting with vague lower urinary tract symptoms and large masses within the pelvis, which makes diagnosis difficult. This rare cancer can easily be misdiagnosed due to the similarity of clinical manifestations to other prostate diseases. A histopathological assessment with the use of immunohistochemistry and molecular diagnostics is still necessary in order to obtain a precise diagnosis. Early multidisciplinary management and systemic treatment could help achieve effective disease control. This particular patient underscores the significance of keeping a high level of suspicion for rare prostatic sarcoma among young patients with unusual presentations.
